# High Genetic Diversity of *Histoplasma* in the Amazon Basin, 2006–2017

**DOI:** 10.3201/eid3106.241386

**Published:** 2025-06

**Authors:** Tani Ly, Marcus de Melo Teixeira, Gaston I. Jofre, Denis Blanchet, Sigrid MacDonald, Primavera Alvarado, Silvia Helena Marques da Silva, Victoria E. Sepúlveda, Qandeel Zeb, Stephen Vreden, Antoine Adenis, Francisco Yegres, Magalie Demar, Maria José Serna Buitrago, Bridget M. Barker, Mathieu Nacher, Daniel R. Matute

**Affiliations:** Université de la Guyane, Cayenne, French Guiana (T. Ly); Universidade de Brasilia, Brasilia, Brazil (M. de Melo Teixeira); Northern Arizona University, Flagstaff, Arizona, USA (M. de Melo Teixeira, B.M. Barker); Virginia Commonwealth University, Richmond, Virginia, USA (G.I. Jofre); Centre Hospitalier de Cayenne, Cayenne (D. Blanchet, A. Adenis); University of Amsterdam, Amsterdam, Netherlands (S. MacDonald); Instituto de Biomedicina, Caracas, Venezuela (P. Alvarado); Evandro Chagas Institute, Ananindeua, Brazil (S.H. Marques da Silva); University of North Carolina, Chapel Hill, North Carolina, USA (V.E. Sepúlveda, Q. Zeb, F. Yegres, D.R. Matute); Foundation of Scientific Research Suriname, Paramaribo, Suriname (S. Vreden); Centre hospitalier Andree Rosemon, Cayenne (M. Demar); Instituto de Salud Carlos III, Madrid, Spain (M.J.S. Buitrago); Centre d'Investigation Clinique Antilles Guyane, Cayenne (M. Nacher).

**Keywords:** Histoplasma, fungi, fungal infections, genetic diversity, Amazon basin, South America, Central America, French Guiana, Brazil

## Abstract

Histoplasmosis is one of the most common pulmonary mycosis diseases in the world. Genome sequencing has revealed that *Histoplasma*, the cause of histoplasmosis, is composed of several phylogenetic species. The genetic diversity of the pathogen remains largely unknown, especially in the tropics. We sequenced the genomes of 91 *Histoplasma* isolates from the Amazon basin of South America and used phylogenomics and population genetic evidence to measure the genetic variation of the genus in South America. We report a previously unidentified clade of *Histoplasma* endemic to the Amazon basin. The lineage is widespread across the continent and contains 5 lineages that are sufficiently differentiated to be considered phylogenetic species. We found the geographic range of those lineages is largely but not completely overlapping. Finally, we found that the patient median age and sex ratio differs among species, suggesting differences in the epidemiology of histoplasmosis caused by each *Histoplasma* lineage.

Fungal diseases have a large and negative effect on human well-being (*1*). Histoplasmosis is one of the most common pulmonary mycosis diseases in the world (*2*). In immunocompetent hosts, cases of histoplasmosis are mildly symptomatic and self resolutive. In those patients, mandatory reporting is not needed, and the disease is often undiagnosed or misdiagnosed. In contrast, histoplasmosis is critical in immunosuppressed patients. In some areas, histoplasmosis will affect up to 25% of the HIV-positive population, and infections frequently are fatal (*3*). Histoplasmosis is diagnosed in ≈500,000 persons each year, and nearly 100,000 persons develop a progressive disseminated disease (*4*). Among persons with advanced HIV (CD4 cell count <200 cells/mm^4^), the disease has a case-fatality rate of <5%–50% when treated (*5*,*6*) and close to 100% if not treated (*6*).

The etiologic agent of histoplasmosis, *Histoplasma*, is a cosmopolitan fungus detected in all continents (*7*) including Antarctica (*8*). Histoplasmin, the main *Histoplasma* antigen, skin testing has revealed the fungus has a large geographic range (*9*). Nuclear gene genealogies and a global sample of *Histoplasma* strains from 8 countries revealed the existence of >7 phylogenetic species (*7*,*10*), monophyletic groups that are reciprocally monophyletic and isolated from each other. Genome sequencing has confirmed the existence of differentiated phylogenetic species within *Histoplasma* (*11*,*12*). Those assessments are limited because the sampling for *Histoplasma* has been heavily biased toward North America and because few samples from other locations have been fully sequenced.

Nonetheless, histoplasmosis is rampant throughout the Americas. Patients in South America suffer from disseminated histoplasmosis at one of the highest incidences in the world (1.5 cases/100 person-years in persons living with HIV) (*13*). Histoplasmin surveys have detected multiple localized foci of high skin reactivity to *Histoplasma* (*14*). Preliminary genetic analysis suggests isolates from South America are genetically diverse (*10*,*15*). More recent approaches have used genomic data and revealed the existence of a phylogenetic species endemic to Rio de Janeiro, Brazil (*16*). Yet, the *Histoplasma* isolates from South America, a continent that is hypothesized as a reservoir of diversity for the genus (*7*,*10*), remain largely uncharacterized genetically. A systematic study of the genome-wide diversity of *Histoplasma* across the Americas is sorely needed. 

In this report, we used whole-genome sequences to measure the genetic diversity of *Histoplasma* across the Americas. We sequenced 91 genomes of isolates from South and Central America, used data from previous sequencing efforts, generated the largest phylogenetic assessment for this pathogen to date (187 genomes), and studied the extent of divergence within *Histoplasma* spp. from the Americas. We identified 5 lineages that meet the classification criteria as phylogenetic species and compared the epidemiology of histoplasmosis caused by each lineage. 

## Materials and Methods

### Fungal Isolates

We obtained pure mycelial cultures from patients in Central and South America who had clinically defined histoplasmosis diagnosed during 2006–2017 and compiled the collection site, sex, age, and the type of sampling and disease for each patient (Appendix Table 1). We subcultured samples on Sabouraud agar with chloramphenicol, gentamycin, and actidione (Bio-Rad Laboratories, https://www.bio-rad.com) to obtain enough fungal biomass for DNA extraction (>500 mg). We then conducted DNA extraction (Appendix).

### Reference Genome for *Histoplasma* mz5-like

We assembled a de novo genome for the *H. suramericanum* strain mz5, an isolate originally collected in Colombia, by using Oxford Nanopore (https://nanoporetech.com) long-reads. We obtained a total of 231,650 reads, with an average length of 4,681.3 bp (National Center for Biotechnology Information Sequence Read Archive accession no. PRJNA1263433). The mean coverage from our reads was 31.13. We used Flye (*17*) to assemble the reads and 3 runs of Racon (*18*) and Medaka (version 1.11.3, https://github.com/nanoporetech/medaka; *19*) to polish the assembly. We used Pilon (*20*) for insertion/deletion corrections 4 times by using the FASTQs files. To assess the quality and completeness of our resulting assembly, we used Quast (*21*) and BUSCO (*22*) with the fungi and Eurotiomycetes OrthoDB V10 databases (*23*). The assembly had 26 contigs; 95.92% of the genome was assembled in the 10 largest contigs, and we focused on those for the phylogenetic analysis.

### Isolate Resequencing

To prepare genomic libraries, we used KAPA library preparation kits (Kapa Biosystems, https://kapabiosystems.com) for Illumina (Illumina, https://www.illumina.com) next-generation sequencing and 1 μg of purified DNA per isolate. Next, we indexed the libraries by using unique 8-bp nucleotide identifiers. We evaluated the concentration of each library with a Kapa library quantification kit (Kapa Biosystems) on a 7900HT Instrument (Thermo Fisher Scientific, https://www.thermofisher.com). We sequenced the libraries to a read length of 100 bp by using v3 or v4 chemistries on an Illumina HiSeq 2500 instrument (Illumina) or to 150 bp by using v2 chemistry on an Illumina NextSeq platform (Illumina), both on a high output mode (paired-end). We recorded the coverage and accession numbers for each isolate (Appendix Table 2).

### Previously Published Data

**Figure 1:**
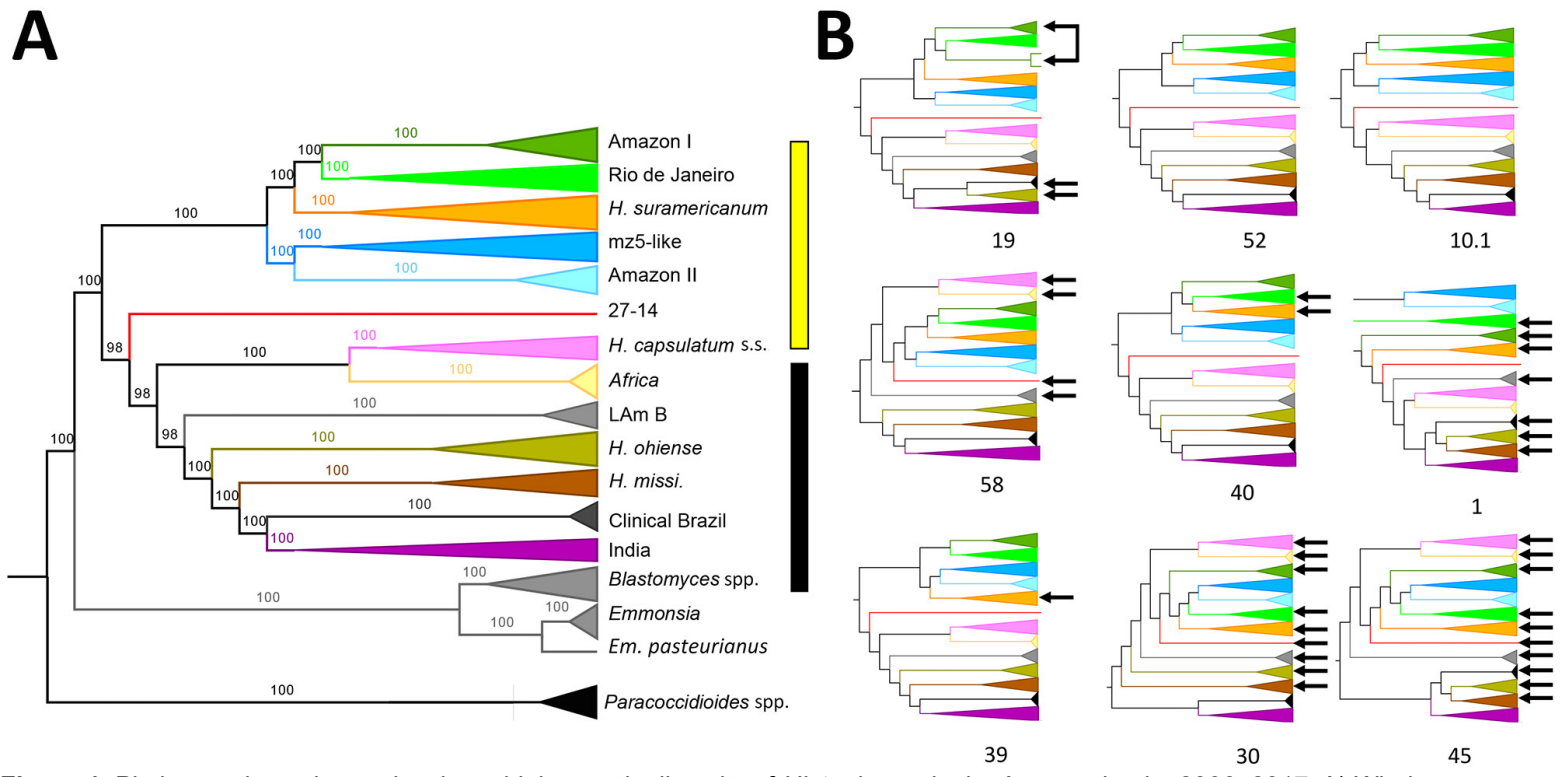
Phylogenetic analyses showing a high genetic diversity of *Histoplasma* in the Amazon basin, 2006–2017. A) Whole-genome concatenated phylogenetic tree with markers shows the existence of >12 monophyletic groups. Yellow bar, the Latin American clade; black bar, the global clade. The numbers above each bar represent the concordance value. B) The 9 largest supercontigs showing largely consistent topologies. Arrows show lineages with positions that differ from the inferred species tree. The identification numbers of each supercontig are below each graph.

To compare the *Histoplasma* isolates from Central and South America with isolates from other locations, we used 30 previously sequenced genomes (*12*). We also used 16 genomes from a *Histoplasma* lineage endemic to India (*24*) and 50 genomes from Rio de Janeiro (*16*). To root the phylogenetic trees (Figure 1), we used *Paracoccidioides* genomes from 2 different species (*P. restrepiensis,* n *=* 3; *P. brasiliensis* sensu stricto, n *=* 2) (*25*), *Blastomyces* (n = 5) (*26*), *Emmonsia crescens* (n = 2) (*27*), and *Emergomyces pasteurianus* (n = 1) (*27*). The genomes all have available Sequence Read Archive accession numbers (Appendix Table 3).

### Read Mapping

We used 1 of the samples from the newly identified Latin America lineage to produce a new reference genome for the group (strain mz5). This reference genome has a total length of 34,827,701 bp, with 26 contigs, an N50 of 5,161,774 bp, and a benchmarking universal single-copy orthologs (BUSCO) completeness assessment of 98.6% complete Eurotiomycetes orthologs and 98.5% complete fungi orthologs (*22*). We mapped 200 short-read sequenced samples (91 newly sequenced genomes) to the reference. The total number of *Histoplasma* genomes from the Americas is 113. We used specific protocols to call variants (Appendix).

### Phylogenetic Analysis

To study the genealogical relationships among *Histoplasma* isolates, we converted our multisample variant call format (VCF) file into a concatenated genome-wide alignment in Phylip format by using a Python script (vcf2phylip, https://zenodo.org/records/2540861). We then extracted the 10 largest contigs from our multisample VCF by using bcftools (*28*). Then, we built maximum likelihood trees from the 10 largest contigs and the genome-wide alignment by using IQ-TREE 2 (*29*). ModelFinder (*30*) determined that the transversion with equal base frequences plus R3 model was the best-fitting model of nucleotide substitution for the genomewide alignment (Appendix Table 4). To estimate branch support, we generated 1,000 tree replicates with an ultrafast bootstrap approximation (*31*). We used a similar approach to generate genealogies for the largest 10 supercontigs in the nuclear genome. We compared those trees by using a Robertson-Foulds distance (*32*) as implemented in the R function treedist (library phangorn) (*33*).

Next, we estimated the extent of genealogical concordance for the nuclear genome in 2 ways. First, we studied whether different genomic windows showed the same genealogy. We split into 325 nonoverlapping windows, each 100 kb, and used IQ-TREE 2 (*29*) to generate a genealogy from each partition (325 genome-window trees; different partitions gave similar results). We calculated the concordance factors (CF) (*34**,**35*) as the fraction of genealogies concordant with each branch from the species trees. Second, we used gene trees from 3,494 complete Eurotiomycetes orthologs (*22**,**23*). Our approach to generate the gene trees is identical to previously described gene genealogies (*12**,**24*). Lineages that showed reciprocal monophyly and had high levels of concordance among supercontigs were treated as potential phylogenetic species for further analyses (*36*).

### Genetic Diversity and Differentiation

We used population genetics approaches to study the partition of genetic variation in *Histoplasma*. We estimated the magnitude of genetic variation (π) within each lineage of *Histoplasma* (as identified by the concatenated phylogenetic tree and the concordance analysis, see immediately above) and compared these values to the magnitude of pairwise divergence between species (Dxy). Instances of advanced speciation show a much higher Dxy between the 2 focal groups than the π value of either group (*37*). We used Pixy (*38*) for all calculations. To compare the values of π in each lineage with the pairwise Dxy, we used an approximative 2-sample Fisher-Pitman permutation test (*39*).

### Patient Characteristics for Each *Histoplasma* Lineage

We studied general epidemiologic patterns of histoplasmosis caused by each lineage identified in this study. For clinical isolates, we collected patient age, sex, and HIV status and a description of the disease (Appendix Table 1). We conducted 3 analyses by using this dataset. First, we compared whether the 4 countries with the largest number of cases (French Guyana, Brazil, Suriname, and Venezuela) had similar proportional representation of the 6 resident *Histoplasma* phylogenetic species by using a 2-sample test for equality of proportions with continuity correction (prop.test function, R library stats) (*40*). We did 6 pairwise comparisons and adjusted the p values by using a Bonferroni correction (The R Project for Statistical Computing, https://www.r-project.org). We calculated the power of the proportion tests by using the function pwr.2p2n.test (R library pwr).

Second, we studied whether reports of histoplasmosis were equally common in males and females across lineages. We used a χ^2^ test by using the R function chisq.test. We calculated the power of each χ^2^ test by using the function power.chisq.test (R library DescTools). We only report the comparisons for the 2 lineages that had a χ^2^ power >0.5. We also compared whether the patient sex proportional representation differed among lineages by using a linear model.

Finally, we compared the age of the histoplasmosis patients affected by the 5 lineages. We used a type-III analysis of variance (R library car) (*40*), followed with Tukey honestly significant difference post-hoc pairwise comparisons (R library multcomp) h (*41*) to identify whether lineages differed from each other.

### Ethics Considerations 

Ethics approval was obtained by the Comité de Protection des Personnes (approval no. CPP2012-47) and the Commission Nationale Informatique et Libertés (approval no. CNIL913511). Biological collection for samples was approved (approval no. DC-2013-1902).

## Results

We used *Histoplasma* samples collected for 11 years (2006–2017) in French Guiana, Suriname, Brazil, Venezuela, Guyana, Martinique, Nicaragua, and Spain to conduct a phylogenetics and population genetics analysis to understand the epidemiologic patterns of histoplasmosis in the Amazon basin and adjacent areas. First, we used the genomewide data to resolve the phylogenetic relationships between *Histoplasma* lineages. We created a maximum-likelihood phylogenetic tree for all samples of *Histoplasma* by using concatenated markers to reveal the existence of a monophyletic group composed of *Histoplasma* from South and Central America (Figure 1, panel A). The clade includes *H. suramericanum*. We refer to this clade as the Latin American *Histoplasma* clade, but the group may contain unsampled lineages from outside Latin America. Most previously sequenced samples from North America, South America, and India (*14*,*24*) form a second monophyletic group, which we refer to as the global *Histoplasma* species complex. The global species complex includes 8 lineages (7 with >1 isolate), 1 from Africa, 1 from India, and 6 from the Americas: *H. ohiense*, *H. mississippiense*, Latin American group B, *H. capsulatum* subspecies (originally thought to be restricted to Central America), and 2 poorly sampled lineages of clinical origin (1 isolate, 27­_14, forming the first lineage and 2 clinical isolates from Brazil forming the second lineage). The 2 clinical isolates were not previously described, but all other lineages have been reported previously (*14*,*24*).

The Latin American lineage only includes samples from South and Central America and is highly diverged from most of the previously sequenced samples of *Histoplasma* from around the world. This lineage contains 5 clades (*H. suramericanum* [*11*], Amazon I, Amazon II, RJ [*16*], and mz5-like). Because this lineage has a mutation rate similar to that of other ascomycetes (*42*), we believe the Latin American lineage is 3.2 million years old (CI 2.3–4.1 million years). 

We evaluated whether the 5 monophyletic groups revealed by the concatenated tree fulfilled the requirements to be considered different phylogenetic species. We tested 2 additional criteria to assess whether the groups were differentiated enough in the speciation continuum. In cases of advanced speciation, different genome sections show consistent evolutionary trajectories. The 5 groups appear as monophyletic in the concatenated analyses, which is consistent with the possibility of each lineage being a phylogenetic species. All the clades, apart from the RJ lineage, co-occur in the Amazon basin. We evaluated whether the signal from the concatenated genome was also consistent at a more granular level (Figure 1, panel B). Local ancestry analyses are consistent with the genomewide results. Concordance between supercontigs was high, but not perfect, and some supercontigs revealed variations in the inferred phylogenetic relationships (Figure 1, panel B; Appendix Figures 1, 2).

CF at the genome window level showed that 4 of the 5 lineages had moderately high CF (CF >50%) (Appendix Figure 1), but 1 group, the lineage Amazon I, showed a low CF of 17%, which suggests diverse genetic trajectories along the genome. Of note, this lineage also showed nonmonophyly in supercontig 19 (Figure 1, panel B). We found the Robertson-Foulds distance between pairs of supercontigs and with the tree inferred from a concatenated alignment (Figure 1, panel A; Appendix Figure 2). In general, recent divergences have high bootstrap support and concordance factors, but older splits had lower support (Figure 1; Appendix Figures 1, 2). Despite those genealogical differences, the genome and local ancestry results indicate a high level of phylogenetic concordance among different genomic regions.

**Figure 2:**
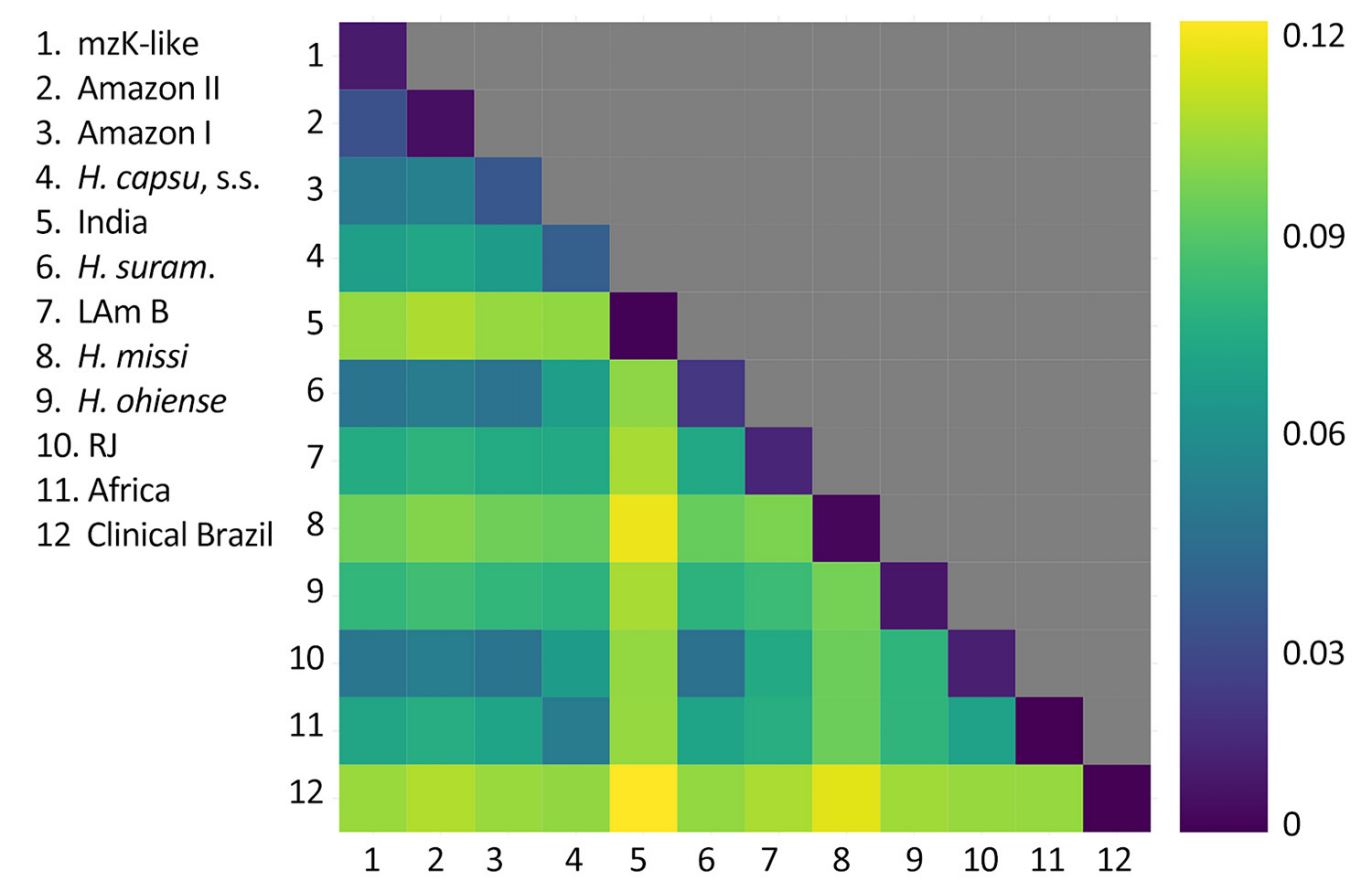
Genetic variation within and between lineages of *Histoplasma* found in the Amazon Basin, 2006–2017. Interspecific genetic distance is larger than the intraspecific variation in most pairwise comparisons. The upper diagonal shows intraspecific variation; all other squares show the pairwise distance between interspecific genetic distance. Unit on right side of graph is pairwise genetic distance.

**Figure 3:**
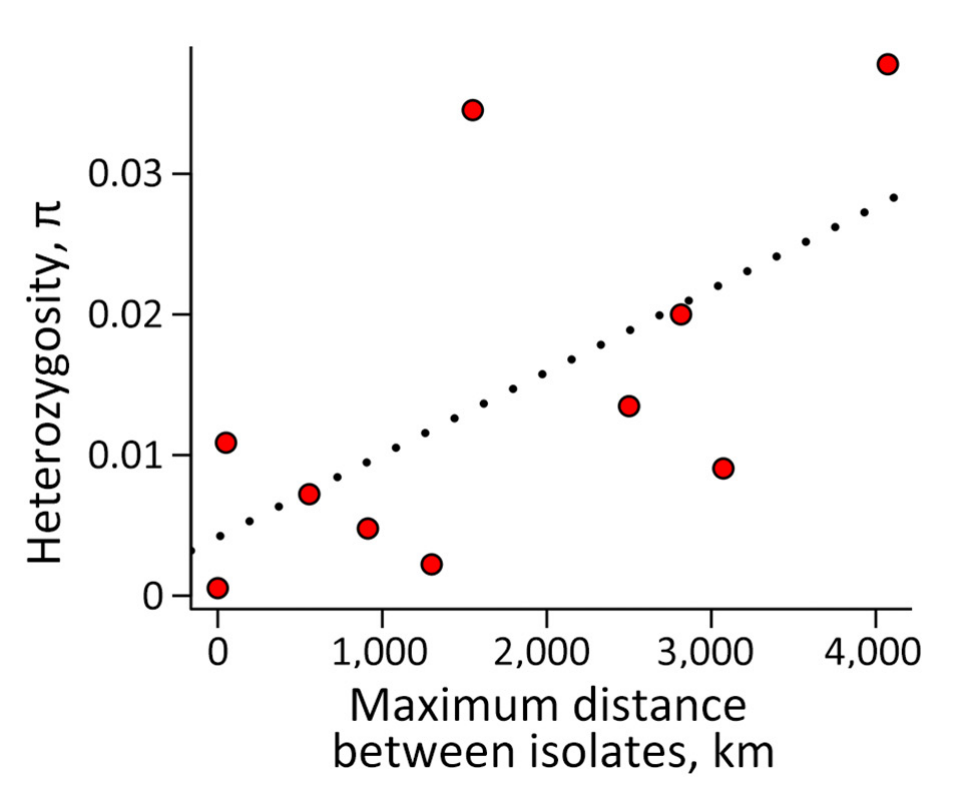
Genetic variation within and between lineages of *Histoplasma* found in the Amazon Basin, 2006–2017. The red dots are the values of nucleotide diversity in each lineage. Dotted line is the predicted linear regression. π is correlated with the geographic range size of each lineage, measured as the distance between the most distant isolates in a lineage. π, within-species genetic variation.

Second, the genetic distance between isolates of different species tends to be larger than the extent of polymorphism within species (*36*,*43*). We evaluated whether the extent of Dxy between the monophyletic lineages was larger than the magnitude of π. We found in almost all pairwise assessments (74 of 78) (Appendix Table 5) that Dxy was larger than the variation in any of the lineages, suggesting increased divergence in Latin America (Figure 2). The 4 pairs of lineages that were not major involved only 2 isolates. Isolate 27_14 was detected in 3 pairs and the clinical isolate from Brazil was detected in 2 pairs, including once in combination with isolate 27­_14 (Appendix Table 5). Of note, we found a weak association between heterozygosity and geographic range size (Spearman rank correlation ρ = 0.685, p = 0.035), indicating the larger ranges might serve as large reservoirs of genetic variation in *Histoplasma* (Figure 3).The extent of genetic differentiation as revealed by both phylogenetic and population genetics approaches suggests that speciation is considerable among the lineages from the Latin American clade and that these lineages fulfill the criteria to be considered phylogenetic species.

**Figure 4:**
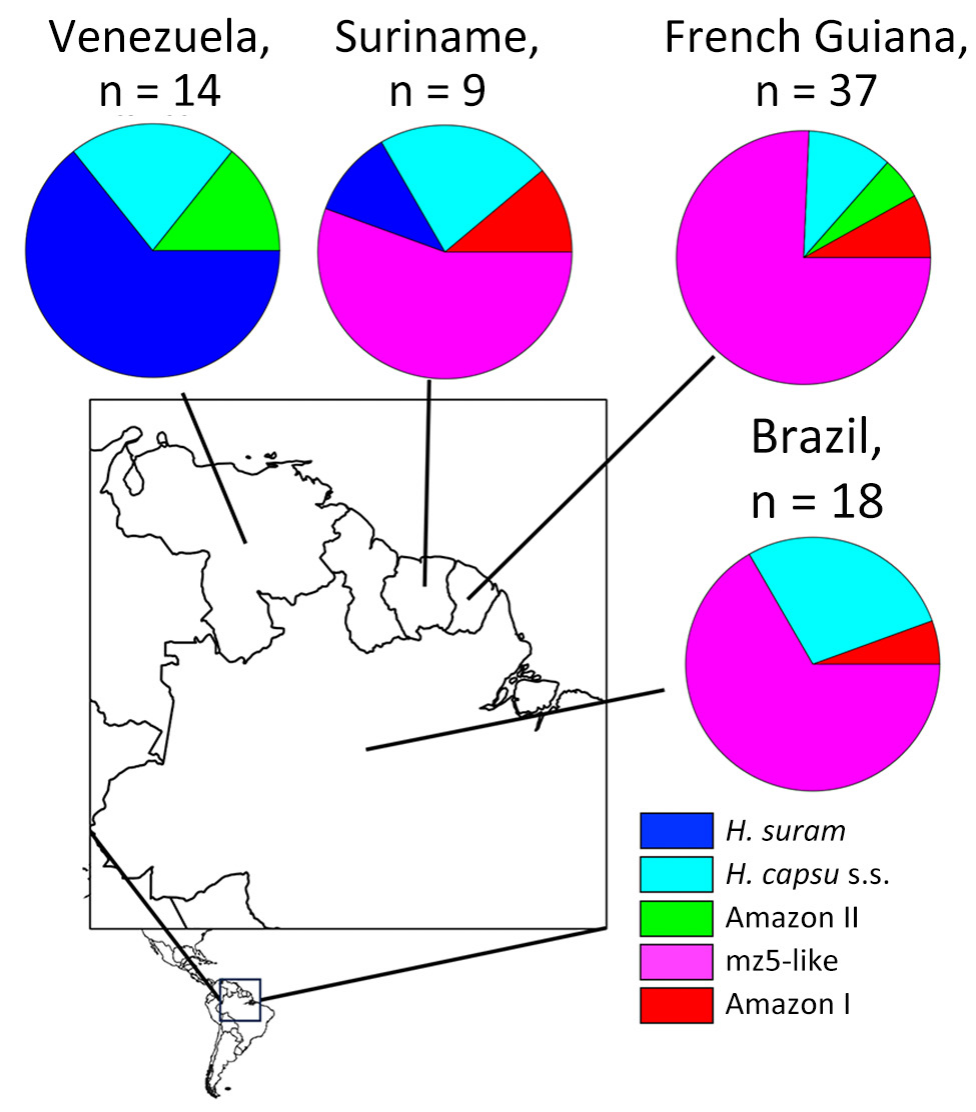
Proportional representation of the *Histoplasma* phylogenetic species identified in 4 countries of the Amazon basin, 2006–2017. s.s., sensu stricto.

We then compared some epidemiologic aspects of histoplasmosis caused by each phylogenetic species. Of the 4 countries that had >10 samples, 3 (Suriname, French Guiana, and Brazil) had a high prevalence of the mz5-like lineage (Figure 4). Venezuela demonstrated a high prevalence of *H. suramericanum* and no mz5-like isolates. Of consequence, the species composition of the Venezuela *Histoplasma* sample is the only sample that differs from other countries (χ^2^ >6.941, degrees of freedom = 1; p<0.008, p_adjusted_<0.034). Of note, the isolates collected from Spain (n = 4) were collected from patients that migrated from South and Central America, and all belong to *H. suramericanum* (Appendix Table 1).

**Figure 5:**
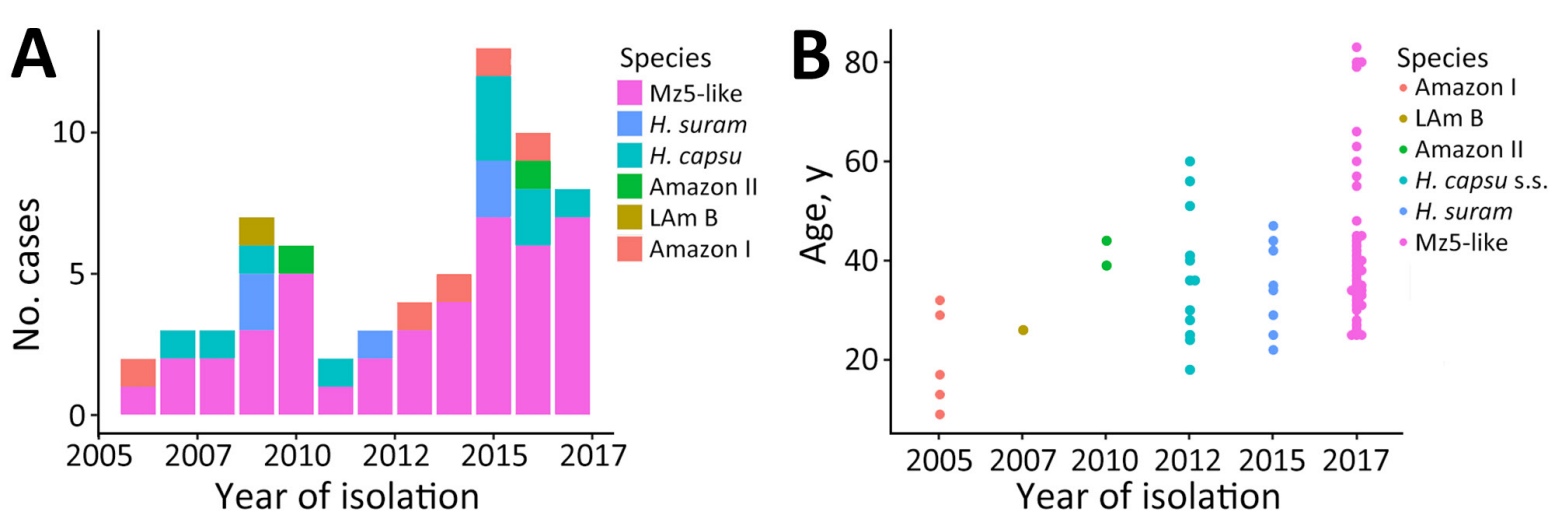
Epidemiologic characteristics of the 6 *Histoplasma* phylogenetic species detected in the Amazon basin, 2006–2017. A) Yearly proportions of each phylogenetic species in the samples collected. B) Age distribution of the patients with histoplasmosis caused by each of the 6 phylogenetic species. LAm B, Latin American group B; s.s., sensu stricto.

We studied whether the species composition of the sample changed between years. We evaluated the proportional representation of each lineage per year (Figure 5, panel A). The year with the highest numbers of cases was 2015 with 13 cases, whereas the lowest were 2005 and 2011 with 2 cases. The mz5-like lineage accounted for >50% of the cases reported in this study in all years except for 2009, when it accounted for 42.9% of the samples. None of those proportions differed significantly from each other (2-sample test for equality of proportions with continuity correction: χ^2^ = 1.641d.f. = 1, p = 0.200).

Third, we compared the mean patient age for each of the lineages identified. We detected significant differences among the age of patients affected by the 5 lineages (1-way analysis of variance: F_5,62_ = 2.621, p = 0.032). Patients with histoplasmosis caused by 4 lineages had a median age of ≈40 years (Figure 5, panel B). Two lineages were significantly different from the others. Amazon I lineage patients had a younger median age (17.0 ± 10.05 years). The mz5-like lineage patients had an older median age (38.0 ± 16.24 years). Amazon II lineage also has a high median age (41.5 years) but a low sample size (n = 2). We performed Tukey pairwise comparisons among lineages (Appendix Table 6).

Finally, we compared the histoplasmosis sex ratio for each of the 6 phylogenetic species. Previous studies have noted that histoplasmosis is more common in men (*44*). All lineages show a higher proportional representation of male than female patients (Table). The mz5-like and *H. capsulatum* s.s. lineages show sufficient power for the comparison (Table). The mz5-like lineage shows no significant differences in the frequency of male and female patients. The *H. capsulatum* subspecies was almost exclusively isolated from male patients (Table). *H. capsulatum* subspecies seems to have a higher male:female patient sex ratio than the other species found in the Amazon basin (Appendix Table 7).

**Table:** The number of cases of histoplasmosis caused by each lineage of histoplasma, the number of patients who are HIV positive, the number of patients with the disseminated form of the disease, and the sex ratio of patients with histoplasma, the amazon basin, 2006–2017*

Lineage	No. cases	HIV positive	Disseminated disease	Sex			χ^2^ power	p value†
				M	F	Ratio		
mz5-like	45	40	25	30	15	2.00	0.918	χ^2^ = 1.931, d.f. = 1, p = 0.165
*H. capsulatum *subspecies	15	11	8	14	1	14.00	0.491	χ^2^ = 4.9658, d.f. = 1, p = 0.026
Amazon I	8	4	3	5	3	1.67	0.293	NA
Amazon II	4	4	2	2	2	2.00	0.232	NA
*H. suramericanum*	15	3	2	6	2	3.00	0.293	NA
Latin American group B	1	1	0	0	1	NA	NA	NA

*d.f., degrees of freedom; NA, not applicable. †By χ^2^ test.

## Discussion

*Histoplasma* is one of the most crucial fungal pathogens in the world, and the disease burden of histoplasmosis is a major concern for public health. Genome sequencing has revealed the existence of multiple cryptic species within the genus (*11*,*14*,*24*). The high incidence of histoplasmosis and the high rates of histoplasmin skin reactivity in South America have led to the hypothesis that *Histoplasma* harbors a high level of genetic diversity in this continent. A potential corollary is this genetic diversity also has clinical implications for patients with histoplasmosis. In this article, we identify a monophyletic group that contains 4 previously unidentified and highly differentiated phylogenetic species endemic to South and Central America, all related to *H. suramericanum*, and demonstrate that South America is a biodiversity hotspot for *Histoplasma* and houses >7 phylogenetic species. The second most diverse continent, to date, is North America with 3 phylogenetic species (*45*).

Our sampling also enabled us to show that the epidemiologic trends of histoplasmosis differ depending on the causal lineage. We report 2 potentially epidemiologic differences of note between lineages of *Histoplasma* spp., the age and sex ratio of the affected patients. Histoplasmosis patients are more frequently male, and the ratio is reported to be close to 3:1 (*14*,*46*). Our findings suggest differences between the *Histoplasma* spp. in the Amazon basin and the sex ratio varies from 3:2 (Amazon I) to 14:1 (*H. capsulatum* s.s.). Nonetheless, those assessments should be considered carefully. The drivers of the HIV epidemic may vary between territories leading to different age distributions and sex ratios. Furthermore, women are usually tested earlier for HIV and are more likely to seek medical care than men. In contrast, outdoor physical labor and exposure to *H. capsulatum* may be more frequent among men. Controlled animal infections will be the ultimate test of whether different lineages of *Histoplasma* represent a different health risk to different sexes.

The identification of phylogenetic species is the first step to understanding the genetic diversity of *Histoplasma* spp. Perhaps the most crucial question is whether different species are associated with differences in virulence and clinical manifestations of histoplasmosis, which remains largely understudied. We report epidemiologic differences among lineages in South America, but only a comparative assessment from multiple isolates from each *Histoplasma* lineage will demonstrate whether genetic differences among lineages also lead to phenotypic differences in clinical traits. Future research should address whether different clades differ in virulence, which in turn will address whether the epidemiologic patterns observed in our study are caused by genetic changes in each of the *Histoplasma* lineages.

Even though South America harbors the highest number of phylogenetic species known in *Histoplasma* to date, our sampling does not enable us to definitively affirm that South America is the most diverse hotspot of *Histoplasma* because sampling on other continents has been limited. We did not name additional species in this study because it is likely there are other groups around the world needing study, and taxonomy should not be revisited until a more global portrait emerges.

Clinical and epidemiologic comparisons between the different lineages of *Histoplasma* remain rare (*14*) (Appendix Table 8) but are a study frontier that can reveal the tempo and mode of evolution of virulence strategies in fungal pathogens. Overall, our results suggest South America is a geographic reservoir of genetic diversity of *Histoplasma* and underscore the need for systematic collection of the agents of endemic mycoses across tropical regions to better understand their evolutionary history. Now that genome sequencing is available for most species, it should be fully deployed to clarify the evolutionary history and epidemiologic patterns of histoplasmosis and other endemic mycoses.

Appendix Additional information about high genetic diversity of *Histoplasma* in the Amazon basin, 2006–2017.
