# Supplementary material for: High Genetic Diversity of Histoplasma in the Amazon Basin, 2006–2017
Source: Emerg Infect Dis. 2025 Jun;31(6):1169–77. doi: 10.3201/eid3106.241386 (PMC12123918; doi:10.3201/eid3106.241386)
Supplement: Appendix — Additional information about high genetic diversity of Histoplasma in the Amazon basin, 2006–2017. [file 24-1386-Techapp-s1.pdf]

Article DOI: https://doi.org/10.3201/eid3106.241386

EID cannot ensure accessibility for supplementary materials supplied by authors. Readers who have difficulty accessing supplementary content should contact the authors for assistance.

High Genetic Diversity of *Histoplasma* in the Amazon Basin, 2006–2017

Appendix

DNA extraction

After subculturing each isolate, we collected ~500 mg of the filamentous phase of *Histoplasma* spp. and heated the tissue at 65°C for 10 min. To extract DNA, we used mechanical lysis by adding 0.5 mm diameter zirconia/silica beads and lysis buffer to the preheated tissue and vigorously agitated the mixture using a Qiagen's Vortex for 15 minutes. To purify DNA, we used the DNAeasy Blood and Tissue Kit (Qiagen, Hilden, Germany) according to the manufactures standard protocol. Finally, we evaluated DNA integrity and concentration using agarose-gel electrophoresis (0.8% w/v) and Qubit fluorimetry (Qubit fluorimeter, Thermo Fisher Scientific, Waltham, MA), respectively.

SNP Variant calling

First, we removed the Illumina adapters from all Illumina reads using Trimmomatic v 0.36 (1). Then, we aligned trimmed reads to the *H. suramericanum* MZ5 reference genome using BWA mem - v 0.7.15 (2). Next, we identify single nucleotide polymorphisms (SNPs) using GATK 4.1.7.0 (3, 4). We used the function HaplotypeCaller setting the -ploidy option as 1 (haploid). We then merged the resulting gvcf files using the GATK GenomicsDBImport function, and jointly genotyped the database with the GATK GenotypeGVCFs function. Finally, we filtered the multisample VCF file using GATK VariantFiltration with the following parameters: QD = 2.0 || FS_filter = 60.0 || MQ_filter = 30.0 || MQ_Rank_Sum_filter = -12.5 || Read_Pos_Rank_Sum_filter = -8.

Appendix Table 1. List of *Histoplasma* spp. isolates used in the study. *capsu*: *H. capsulatum sensu stricto. sura*: *H. suramericanum*

Isolate Name	Clade	Country of isolation	Source	Sex	Age	Country of birth	Nature of sampling	Disseminated /localized	Year of isolation
FG-pia2052	mz5-like	French Guiana	Human	f	83	French Guiana	arthritis	arthritis	2009
FG-pir2086	mz5-like	French Guiana	Human	m	79	NA	tongue	disseminated	2009
FG-ama2041	mz5-like	French Guiana	Human/HIV+	m	25	Guyana	bone marrow	NA	2010
FG-bel2002	mz5-like	French Guiana	Human/HIV+	m	55	Haiti	blood	disseminated	2017
FG-bik2051	mz5-like	French Guiana	Human/HIV+	f	38	French Guiana	ganglion	disseminated	2017
FG-bon2001	mz5-like	French Guiana	Human/HIV+	m	44	Suriname	bone marrow	disseminated	2015
FG-cle2015	mz5-like	French Guiana	Human/HIV+	m	57	Haiti	liver	disseminated	2012
FG-cri2041	mz5-like	French Guiana	Human/HIV+	m	66	Saint-Lucia	Stomach	digestive	2010
FG-das2062	mz5-like	French Guiana	Human/HIV+	f	38	Brazil	colon	disseminated	2013
FG-dee2016	mz5-like	French Guiana	Human/HIV+	f	33	Suriname	bone marrow	digestive	2010
FG-deo2088	mz5-like	French Guiana	Human/HIV+	f	39	Brazil	cerebrospinal fluid	disseminated	2014
FG-fan2059	mz5-like	French Guiana	Human/HIV+	m	45	Suriname	Lymph node	ganglionic and colic	2017
FG-fer2036	mz5-like	French Guiana	Human/HIV+	m	43	Brazil	bone marrow	disseminated	2017
FG-gre 2022	mz5-like	French Guiana	Human/HIV+	f	40	Haiti	blood	disseminated	2016
FG-jos2044	mz5-like	French Guiana	Human/HIV+	m	40	French Guiana	colon	disseminated	2016
FG-kou2024	mz5-like	French Guiana	Human/HIV+	m	26	NA	blood	NA	2017
FG-lin2055	mz5-like	French Guiana	Human/HIV+	m	63	French Guiana	Bronchoalveolar lavage	NA	2014
FG-mel2036	mz5-like	French Guiana	Human/HIV+	f	41	Brazil	liver	hepatic	2007
FG-non2028	mz5-like	French Guiana	Human/HIV+	m	60	Brazil	urine	disseminated	2017
FG-pie2055	mz5-like	French Guiana	Human/HIV+	m	28	NA	bone marrow	NA	2017
FG-pin2043	mz5-like	French Guiana	Human/HIV+	m	34	NA	colon	digestive	2010
FG-poe2043	mz5-like	French Guiana	Human/HIV+	f	35	NA	colon	NA	2012
FG-rod2046	mz5-like	French Guiana	Human/HIV+	m	32	Brazil	bone marrow	disseminated	2010
FG-sou0318	mz5-like	French Guiana	Human/HIV+	m	34	Brazil	blood	disseminated	2006
FG-ver2032	mz5-like	French Guiana	Human/HIV+	f	33	Suriname	ganglion	NA	2009
FG-zaa2004	mz5-like	French Guiana	Human/HIV+	f	30	Suriname	bone marrow	disseminated	2007
FG-zul2036	mz5-like	French Guiana	Human/HIV+	f	45	French Guiana	subclavicule	disseminated	2009
S-ada2068	mz5-like	Suriname	Human/HIV+	f	35	Suriname	bone marrow	NA	2014
S-asa2073	mz5-like	Suriname	Human/HIV+	m	37	Suriname	bone marrow	NA	2014
S-asaazp1	mz5-like	Suriname	Human/HIV+	m	NA	Suriname	blood	NA	2013
S-mis2065	mz5-like	Suriname	Human/HIV+	m	27	Suriname	bone marrow	NA	2014
S-spa2057	mz5-like	Suriname	Human/HIV+	f	34	Suriname	liver	NA	2015

Isolate Name	Clade	Country of isolation	Source	Sex	Age	Country of birth	Nature of sampling	Disseminated /localized	Year of isolation
FG-ada2079	mz5-like	French Guiana	Human/HIV+	m	48	French Guiana	bone marrow	disseminated	2013
HCAM	Amazon_I	Venezuela	Human/HIV+	f	NA	Venezuela	bone marrow	disseminated	NA
HCM-H	Amazon_I	Venezuela	Human/HIV+	m	NA	Venezuela	NA	disseminated	NA
FG-bre2013	Amazon_I	French Guiana	Human/HIV+	m	39	Saint-Lucia	bone marrow	NA	2010
FG-pic2055	Amazon_I	French Guiana	Human/HIV+	f	44	French Guiana	ganglion	ganglionic	2016
FG-aco2005	Amazon_II	French Guiana	Human	m	9	French Guiana	blood	disseminated	2013
FG-bru	Amazon_II	French Guiana	Human/HIV+	f	32	Cayenne	blood	NA	2006
FG-pers2034	Amazon_II	French Guiana	Human/HIV+	m	29	French Guiana	bone marrow	disseminated	2016
S-dor2042	Amazon_II	Suriname	Human/HIV+	f	17	Suriname	NA	NA	2014
S-dij2058	*sura*	Suriname	Human/HIV+	m	34	Suriname	bone marrow	NA	2015
HC7072a	*sura*	Venezuela	Human			Venezuela		NA	NA
HC7090	*sura*	Venezuela	Human/HIV+	m	25	Venezuela	skin	NA	NA
HC970591	*sura*	Venezuela	Human/HIV+	f	29	Venezuela	lung	NA	NA
hcjb	*sura*	Venezuela	Human	m	44	Venezuela		disseminated	NA
HC1070058-2	*sura*	Venezuela	Human	m	22	Venezuela		disseminated	NA
HC4137	*sura*	Venezuela	Human	f	42	Venezuela	skin	NA	NA
HC3066	*sura*	Venezuela	Human	NA	NA	NA	NA	NA	NA
HC776	*sura*	Venezuela	Human	m	35	Venezuela	erythematous plaques	NA	NA
HC394	*sura*	Venezuela	Human	m	47	Venezuela	erythematous plaques	NA	NA
B06379	*sura*	Nicaragua	Soil	NA	NA	NA	NA	NA	NA
CM5679	*sura*	Spain	Human	NA	NA	Mexico	NA	NA	2009
CM5692	*sura*	Spain	Human	NA	NA	Mexico	NA	NA	2009
CM7057	*sura*	Spain	Human	NA	NA	Mexico	NA	NA	2012
CM7717	*sura*	Spain	Human	NA	NA	Mexico	NA	NA	2015
A-290302130	LAmB	Martinique	Human/HIV+ ?	f	26	Martinique	Bronchial aspiration	NA	2009
HCR-P	*capsu*	Venezuela	Human	m	25	Venezuela		NA	NA
HC3645	*capsu*	Venezuela	Human/HIV+	m	NA	NA	Nasal granuloma	NA	NA
HC970588	*capsu*	Venezuela	Human	m	18	Venezuela	NA	disseminated	NA
FG-sat2037	*capsu*	French Guiana	Human	m	36	NA	ascites	disseminated	2009
FG-dos0487	*capsu*	French Guiana	Human/HIV+	m	24	NA	bone marrow	NA	2007
FG-martin	*capsu*	French Guiana	Human/HIV+	m	56	Brazil	blood	disseminated	2017
FG-wil2021	*capsu*	French Guiana	Human/HIV+	m	30	French Guiana	blood	disseminated	2008
G-JJ	*capsu*	Republic of Guyana	Human/HIV+	m	40	Republic of Guyana	bone marrow	disseminated	2015
S-rob2039	*capsu*	Suriname	Human/HIV+	m	28	Suriname	blood	NA	2014
S-tan2075	*capsu*	Suriname	Human/HIV+	m	51	Suriname	blood	NA	2014
11571 (Belem 1)	mz5-like	Brazil	Human	M	80	Brazil	ganglion	NA	2008
4363 (Belem 10)	mz5-like	Brazil	Human/HIV+	F	42	Brazil	bone marrow	Disseminated	2015
	mz5-like	Brazil		M	25	Brazil		Disseminated	2015

Isolate Name	Clade	Country of isolation	Source	Sex	Age	Country of birth	Nature of sampling	Disseminated /localized	Year of isolation
4107 (Belem 11)			Human/HIV+				bone marrow		
4516 (Belem 12)	Amazon_III	Brazil	Human/HIV+	M	13	Brazil	bone marrow	Disseminated	2015
4809 (Belem 13)	capsu	Brazil	Human/HIV+	M		Brazil	bone marrow	NA	2016
47692 (Belem 14)	mz5-like	Brazil	Human/HIV+	M	25	Brazil	Urine	Disseminated	2016
4265 (Belem 15)	mz5-like	Brazil	Human/HIV+	M	36	Brazil	bone marrow	Disseminated	2015
4729 (Belem 16)	mz5-like	Brazil	Human/HIV+	M	31	Brazil	Urine	Disseminated	2016
4337 (Belem 17)	mz5-like	Brazil	Human/HIV+	M	31	Brazil	bone marrow	Disseminated	2015
5215 (Belem 18)	mz5-like	Brazil	Human/HIV+	M	NA	Brazil	bone marrow	Disseminated	2016
52292 (Belem 19)	*capsu*	Brazil	Human/HIV+	M	NA	Brazil	bone marrow	Disseminated	2016
11572 (Belem 2)	mz5-like	Brazil	Human	M	80	Brazil	Ganglion	NA	2008
5254 (Belem 20)	mz5-like	Brazil	Human/HIV+	F	NA	Brazil	bone marrow	Disseminated	2016
2202 (Belem 5)	mz5-like	Brazil	Human/Transplant	M	NA	Brazil	Bronchial aspiration	NA	2011
2353 (Belem 6)	*capsu*	Brazil	Human	M	41	Brazil	Lung biopsy	Pulmonary	2011
3948 (Belem 7)	mz5-like	Brazil	Human/HIV+	M	NA	Brazil	bone marrow	Disseminated	2015
3865 (Belem 8)	*capsu*	Brazil	Human/HIV+	F	36	Brazil	bone marrow	Disseminated	2015
4182 (Belem 9)	*capsu*	Brazil	Human/HIV+	M	60	Brazil	bone marrow	Disseminated	2015

Appendix Table 2. SRA for sequences generated in this study.

Isolate Name	SRA Accession number
A-290302130	SRR31893250
B06379	SRR31893266
CM5679	SRR31893265
CM5692	SRR31893264
CM7057	SRR31893263
CM7717	SRR31893262
FG-aco2005	SRR31893282
FG-ada2079	SRR31893313
FG-ama2041	SRR31893312
FG-bel2002	SRR31893301
FG-bik2051	SRR31893289
FG-bon2001	SRR31893279
FG-bre2013	SRR31893285
FG-bru0291	SRR31893281
FG-cle2015	SRR31893268
FG-cri2041	SRR31893257
FG-das2062	SRR31893249
FG-dee2016	SRR31893248
FG-deo2088	SRR31893247
FG-dos0487	SRR31893277
FG-fan2059	SRR31893311
FG-fer2036	SRR31893310
FG-gre 2022	SRR31893309
FG-jos2044	SRR31893308
FG-kou2024	SRR31893307
FG-lin2055	SRR31893306
FG-martin	SRR31893276
FG-mel2036	SRR31893305
FG-non2028	SRR31893304
FG-pers2034	SRR31893280
FG-pia2052	SRR31893303
FG-pic2055	SRR31893284
FG-pie2055	SRR31893302
FG-pin2043	SRR31893300
FG-pir2086	SRR31893299
FG-poe2043	SRR31893298
FG-rod2046	SRR31893296
FG-sat2037	SRR31893275
FG-sou0318	SRR31893295
FG-ver2032	SRR31893293
FG-wil2021	SRR31893274
FG-zaa2004	SRR31893286
FG-zul2036	SRR31893292
G-JJ	SRR31893273
HC1070058-2	SRR31893258
HC3066	SRR31893256
HC3645	SRR31893270
HC394	SRR31893255
HC4137	SRR31893254
HC7072a	SRR31893261
HC7090	SRR31893260
HC776	SRR31893253
HC970588	SRR31893269
HC970591	SRR31893252
HCAM	SRR31893287
hcjb	SRR31893251
HCM-H	SRR31893283
HCR-P	SRR31893267
S-ada2068	SRR31893291
S-asa2073	SRR31893297
S-asaazp1	SRR31893290
S-dij2058	SRR31893259
S-dor2042	SRR31893278
S-mis2065	SRR31893294
S-rob2039	SRR31893272
S-spa2057	SRR31893288
S-tan2075	SRR31893271

Appendix Table 3. SRA accession numbers for accession previously sequenced and used in this study.

Isolate name	SRA accession number	Phylogenetic species	Reference
104_p_06_S19	SRR27481878	India	(5)
104_P_19_S5	SRR27481877	India	(5)
107_P_06_S1	SRR27481870	India	(5)
117_p_12_S17	SRR27481869	India	(5)
122_p_10_B_S15	SRR27481868	India	(5)
136_P_07_S6	SRR27481867	India	(5)
144_p_08_S14	SRR27481866	India	(5)
1517_p_17_S20	SRR27481865	India	(5)
256_P_18_S2	SRR27481864	India	(5)
316_p_10_S18	SRR27481863	India	(5)
327_P_12_S7	SRR27481876	India	(5)
343_p_18_S1	SRR27481875	India	(5)
388_p_11_S16	SRR27481874	India	(5)
S11-105_p_06	SRR27481873	India	(5)
S14-108_p_06	SRR27481872	India	(5)
S16-106_p_06	SRR27481871	India	(5)
4	SRR8084704	RJ	(6)
3	SRR8084705	RJ	(6)
2	SRR8084706	RJ	(6)
1	SRR8084707	RJ	(6)
8	SRR8084708	RJ	(6)
7	SRR8084709	RJ	(6)
6	SRR8084710	RJ	(6)
12	SRR8084714	RJ	(6)
11	SRR8084715	RJ	(6)
14	SRR8084716	RJ	(6)
13	SRR8084717	RJ	(6)
15	SRR8084719	RJ	(6)
18	SRR8084720	RJ	(6)
17	SRR8084721	RJ	(6)
G222B	SRX3350818	*H. ohiense*	(7)
G217B	SRX3350817	*H. ohiense*	(7)
CI_10	SRX3350821	*H. ohiense*	(7)
CI_4	SRX3350841	*H. ohiense*	(7)
CI_17	SRX3350822	*H. ohiense*	(7)
CI_9	SRX3350820	*H. ohiense*	(7)
CI_30	SRX3350824	*H. ohiense*	(7)
CI_18	SRX3350823	*H. ohiense*	(7)
CI_6	SRX3350819	*H. ohiense*	(7)
CI_35	SRX3350825	*H. ohiense*	(7)
CI_24	SRX3350845	*H. mississippiense*	(7)
CI_43	SRX3350837	*H. mississippiense*	(7)
CI_22	SRX3350842	*H. mississippiense*	(7)
CI_7	SRX3350840	*H. mississippiense*	(7)
CI_42	SRX3350844	*H. mississippiense*	(7)
505	SRX3350830	*H. mississippiense*	(7)
DOWNS	SRX3350816	*H. mississippiense*	(7)
CI_19	SRX3350843	*H. mississippiense*	(7)
WU24	SRX3350838	*H. mississippiense*	(7)
UCLA_531	SRX3350836	*H. mississippiense*	(7)
21_14	SRX3350835	*H. suramericanum*	(7)
3_11G	SRX3350833	*H. suramericanum*	(7)
27_14	SRX3350832	*H. suramericanum*	(7)
1986	SRX3350827	*H. capsulatum ss*	(7)
MZ5	SRX3350829	*H. capsulatum ss*	(7)
G186A	SRX3350828	*H. capsulatum ss*	(7)
G184A	SRX3350831	*H. capsulatum ss*	(7)
MV3	SRX3350826	N/D	(7)
duboisii_A	SRX3350834	Africa	(7)
duboisii_B	SRX3350839	Africa	(7)
109_P_06_S4	PRJNA1201237	*B. dermatitidis*	(8)
143_P_08_S8	PRJNA1201237	*B. dermatitidis*	(8)
Dr_Anuradha_Fungal_WGS_S13	PRJNA1201237	*B. dermatitidis*	(8)
Ep_130_s_7	PRJNA1201237	*B. parvus*	(9)
ep139_s_1	PRJNA178178	*B. silverae* (previously classified as *B. parvus*)	(9)
Pb_339	SRR4024750	*P. restrepiensis*	(10)

Isolate name	SRA accession number	Phylogenetic species	Reference
Pb_60855	SRR4024748	*P. restrepiensis*	(10)
Pb_66_ATCACG_L001	SAMN05171529	*P. brasiliensis*	(10)
Pb_jam	SRR4024745	*P. restrepiensis*	(10)
PbD02_TAGCTT_L001	SRR4024744	*P. brasiliensis*	(10)
Ep_9510_s_8	PRJNA416769	*E. pasteurianus*	(9)
Ec_4076_s_7	PRJNA178252	*E. crescens*	(9)
s_2	PRJNA178252	*E. crescens*	(9)

Appendix Table 4. Modeltest results showing the fit for a variety of molecular evolution models.

Model	LogLik	df	AIC	AICc	BIC
TVMe+R3	103668491	357	207337695	207337695	207342619
SYM+R3	103668573	358	207337863	207337863	207342800
TVM+F+R3	103670318	360	207341356	207341357	207346321
GTR+F+R3	103670417	361	207341557	207341557	207346536
GTR+F+R4	103670408	363	207341542	207341542	207346548
TIM3e+R3	103689435	356	207379583	207379583	207384493
TIM2e+R3	103690032	356	207380777	207380777	207385686
TPM3+F+R3	103695571	358	207391858	207391859	207396796
TPM3u+F+R3	103695571	358	207391858	207391859	207396796
TIM3+F+R3	103695572	359	207391861	207391861	207396813
K3P+R3	103696528	355	207393766	207393766	207398662
TIMe+R3	103696527	356	207393765	207393765	207398675
TPM2u+F+R3	103696580	358	207393877	207393877	207398814
TPM2+F+R3	103696580	358	207393877	207393877	207398814
TIM2+F+R3	103696589	359	207393897	207393897	207398848
K2P+R3	103702989	354	207406685	207406685	207411568
TNe+R3	103702987	355	207406685	207406685	207411581
K3Pu+F+R3	103707149	358	207415014	207415014	207419951
TIM+F+R3	103707149	359	207415016	207415016	207419967
HKY+F+R3	103713799	357	207428311	207428311	207433235
TN+F+R3	103713799	358	207428313	207428313	207433250
GTR+F+R2	104383577	359	208767872	208767872	208772823
GTR+F+G4	106154285	358	212309285	212309285	212314223
GTR+F+I+G4	106154285	359	212309287	212309287	212314238
GTR+F	112489650	357	224980015	224980015	224984938
GTR+F+I	112489657	358	224980031	224980031	224984968
JC+R3	116591713	353	233184131	233184131	233189000
F81+F+R3	116614415	356	233229541	233229542	233234451

Appendix Table 5. Fisher Pittman permutation comparing values of intraspecific heterozygosity across *Histoplasma* species.

Species 1	Species 2	$\bar{\pi}$Species1	$\bar{\pi}$Species2	Dxy	Z value	P value
Amazon II	Amazon I	0.00478	0.00905	0.03139	-36.286	< 0.0001
Amazon III	Amazon I	0.03453	0.00905	0.05077	-38.201	< 0.0001
Amazon III	Amazon II	0.03453	0.00478	0.05526	-6.753	< 0.0001
H. capsulatum	Amazon I	0.03779	0.00905	0.07106	-47.530	< 0.0001
H. capsulatum	Amazon II	0.03779	0.00478	0.07565	-17.518	< 0.0001
H. capsulatum	Amazon III	0.03779	0.03453	0.06946	-19.785	< 0.0001
Clinical	Amazon I	0.00014	0.00905	0.10753	-34.894	< 0.0001
Clinical	Amazon II	0.00014	0.00478	0.11182	-4.470	< 0.0001
Clinical	Amazon III	0.00014	0.03453	0.10742	-6.340	< 0.0001
Clinical	*H. capsulatum*	0.00014	0.03779	0.10603	-16.492	< 0.0001
Clinical	India	0.00014	0.00054	0.12647	-13.038	< 0.0001
Clinical	LAm A	0.00014	0.02000	0.10612	-12.643	< 0.0001
Clinical	LAm B	0.00014	0.01348	0.11052	-2.990	0.00410
Clinical	*H. mississippiense*	0.00014	0.00223	0.12098	-8.062	< 0.0001
Clinical	*H. ohiense*	0.00014	0.00722	0.10857	-8.768	< 0.0001
Clinical	Rio	0.00014	0.01088	0.10695	-12.250	< 0.0001
Clinical	27-14	0.00014	NA	0.11063	-1.414	0.33523
Clinical	*H. capsulatum var. duboisii*	0.00014	0.00020	0.10672	-2.236	0.06961
H. capsulatum var. duboisii	Amazon I	0.00020	0.00905	0.07463	-34.783	< 0.0001
H. capsulatum var. duboisii	Amazon II	0.00020	0.00478	0.07908	-4.468	< 0.0001

Species 1	Species 2	$\bar{\pi}$Species1	$\bar{\pi}$Species2	Dxy	Z value	P value
H. capsulatum var. duboisii	Amazon III	0.00020	0.03453	0.07374	-5.795	< 0.0001
H. capsulatum var. duboisii	*H. capsulatum*	0.00020	0.03779	0.05283	-10.268	< 0.0001
H. capsulatum var. duboisii	India	0.00020	0.00054	0.10668	-13.038	< 0.0001
H. capsulatum var. duboisii	LAm A	0.00020	0.02000	0.07387	-12.113	< 0.0001
H. capsulatum var. duboisii	LAm B	0.00020	0.01348	0.07933	-2.979	0.00460
H. capsulatum var. duboisii	*H. mississippiense*	0.00020	0.00223	0.09830	-8.061	< 0.0001
H. capsulatum var. duboisii	*H. ohiense*	0.00020	0.00722	0.08252	-8.762	< 0.0001
H. capsulatum var. duboisii	Rio	0.00020	0.01088	0.07301	-12.146	< 0.0001
H. capsulatum var. duboisii	27-14	0.00020	NA	0.07774	-1.414	0.33043
India	Amazon I	0.00054	0.00905	0.10666	-45.523	< 0.0001
India	Amazon II	0.00054	0.00478	0.11112	-15.163	< 0.0001
India	Amazon III	0.00054	0.03453	0.10674	-16.924	< 0.0001
India	*H. capsulatum*	0.00054	0.03779	0.10588	-26.423	< 0.0001
LAm A	Amazon I	0.02000	0.00905	0.04883	-44.606	< 0.0001
LAm A	Amazon II	0.02000	0.00478	0.05255	-13.595	< 0.0001
LAm A	Amazon III	0.02000	0.03453	0.04810	-14.389	< 0.0001
LAm A	*H. capsulatum*	0.02000	0.03779	0.07045	-25.531	< 0.0001
LAm A	India	0.02000	0.00054	0.10524	-23.280	< 0.0001
LAm B	Amazon I	0.01348	0.00905	0.07746	-35.558	< 0.0001
LAm B	Amazon II	0.01348	0.00478	0.08173	-5.179	< 0.0001
LAm B	Amazon III	0.01348	0.03453	0.07690	-6.702	< 0.0001
LAm B	*H. capsulatum*	0.01348	0.03779	0.07663	-16.293	< 0.0001
LAm B	India	0.01348	0.00054	0.11009	-13.738	< 0.0001
LAm B	LAm A	0.01348	0.02000	0.07606	-13.066	< 0.0001
H. mississippiense	Amazon I	0.00223	0.00905	0.09872	-40.593	< 0.0001
H. mississippiense	Amazon II	0.00223	0.00478	0.10309	-10.195	< 0.0001
H. mississippiense	Amazon III	0.00223	0.03453	0.09885	-11.829	< 0.0001
H. mississippiense	*H. capsulatum*	0.00223	0.03779	0.09737	-21.640	< 0.0001
H. mississippiense	India	0.00223	0.00054	0.12312	-18.707	< 0.0001
H. mississippiense	LAm A	0.00223	0.02000	0.09736	-18.326	< 0.0001
H. mississippiense	LAm B	0.00223	0.01348	0.10176	-8.764	< 0.0001
H. ohiense	Amazon I	0.00722	0.00905	0.08335	-41.301	< 0.0001
H. ohiense	Amazon II	0.00722	0.00478	0.08770	-10.900	< 0.0001
H. ohiense	Amazon III	0.00722	0.03453	0.08321	-12.436	< 0.0001
H. ohiense	*H. capsulatum*	0.00722	0.03779	0.08156	-21.764	< 0.0001
H. ohiense	India	0.00722	0.00054	0.11017	-19.398	< 0.0001
H. ohiense	LAm A	0.00722	0.02000	0.08156	-18.951	< 0.0001
H. ohiense	LAm B	0.00722	0.01348	0.08617	-9.473	< 0.0001
H. ohiense	*H. mississippiense*	0.00722	0.00223	0.09995	-14.443	< 0.0001
Rio	Amazon I	0.01088	0.00905	0.04946	-44.670	< 0.0001
Rio	Amazon II	0.01088	0.00478	0.05339	-14.207	< 0.0001
Rio	Amazon III	0.01088	0.03453	0.04855	-14.767	< 0.0001
Rio	*H. capsulatum*	0.01088	0.03779	0.06933	-24.148	< 0.0001
Rio	India	0.01088	0.00054	0.10625	-22.865	< 0.0001
Rio	LAm A	0.01088	0.02000	0.04731	-21.328	< 0.0001
Rio	LAm B	0.01088	0.01348	0.07667	-12.910	< 0.0001
Rio	*H. mississippiense*	0.01088	0.00223	0.09826	-17.922	< 0.0001
Rio	*H. ohiense*	0.01088	0.00722	0.08248	-18.634	< 0.0001
27-14	Amazon I	NA	0.00905	0.07236	-33.879	< 0.0001
27-14	Amazon II	NA	0.00478	0.07637	-3.738	0.00030
27-14	Amazon III	NA	0.03453	0.07133	-4.858	< 0.0001
27-14	*H. capsulatum*	NA	0.03779	0.07572	-13.386	< 0.0001
27-14	India	NA	0.00054	0.10949	-12.329	< 0.0001
27-14	LAm A	NA	0.02000	0.06717	-10.551	< 0.0001
27-14	LAm B	NA	0.01348	0.08205	-2.235	0.10051
27-14	*H. mississippiense*	NA	0.00223	0.10127	-7.347	< 0.0001
27-14	*H. ohiense*	NA	0.00722	0.08541	-8.047	< 0.0001
27-14	Rio	NA	0.01088	0.07068	-11.304	< 0.0001

Appendix Table 6. Pairwise comparisons reveal a difference in patient age among *Histoplasma* species in the Amazon basin. All comparisons were done with a post-hoc Tukey test (with multiple comparison corrections). All t-tests were done with 62 degrees of freedom (i.e., the number of residual degrees of freedom in a One-way ANOVA).

**Hypothesis**	**Estimate**	**Std. Error**	**t value**	**Pr(>|t|)**
LAmB - Amazon I == 0	6.000	15.986	0.375	0.9988
Amazon II - Amazon I == 0	21.500	12.210	1.761	0.4608
H. capsu - Amazon I == 0	17.083	7.768	2.199	0.2246
LAmA - Amazon I == 0	14.750	8.319	1.773	0.4532
mz5-like - Amazon I == 0	22.925	6.922	3.312	0.0155 *
AmazonII - LAmB == 0	15.500	17.873	0.867	0.9445
H. capsu - LAmB == 0	11.083	15.189	0.730	0.9732
H. suram - LAmB == 0	8.750	15.478	0.565	0.9914
mz5-like - LAmB == 0	16.925	14.775	1.146	0.8400
H. capsu - Amazon_II == 0	-4.417	11.146	-0.396	0.9984
H. suram - Amazon_II == 0	-6.750	11.537	-0.585	0.9900
mz5-like - Amazon_II == 0	1.425	10.574	0.135	1.0000
H. suram - *H. capsu* == 0	-2.333	6.661	-0.350	0.9991
mz5-like - *H. capsu* == 0	5.842	4.803	1.216	0.8039
mz5-like - *H. suram* == 0	8.175	5.652	1.446	0.6662

Appendix Table 7. Binomial regression coefficients comparing the patient sex ratio for the *Histoplasma* phylogenetic species present in the Amazon basin.

Characteristic	Estimate	Std. Error	z value	Pr(>|z|)
Intercept	-1.995e-15	1.000	0.000	1.000
Amazon I	-0.511	1.238	-0.413	0.680
H. capsulatum ss.	2.639	1.439	-1.834	0.066
LAmB	0.156	1,455.00	0.011	0.992
mz5-like	-0.693	1.049	-0.661	0.509
H. suramericanum	0.154	1.144	0.135	0.893

Appendix Table 8. Clinical and epidemiological data availability for the different phylogenetic species of *Histoplasma*. We only included studies that have used whole genome data.

**Study**	**Species included in the study**	**Clinical and epidemiological data available**
(7)	*H. ohiense, H. mississippiensis, H. suramericanum*, *H. capsulatum sensu stricto*, Africa	None
(5)	India	None
(3)	*H. ohiense, H. mississippiensis, H. suramericanum*	None
(6)	RJ	Yes, for the RJ lineage.

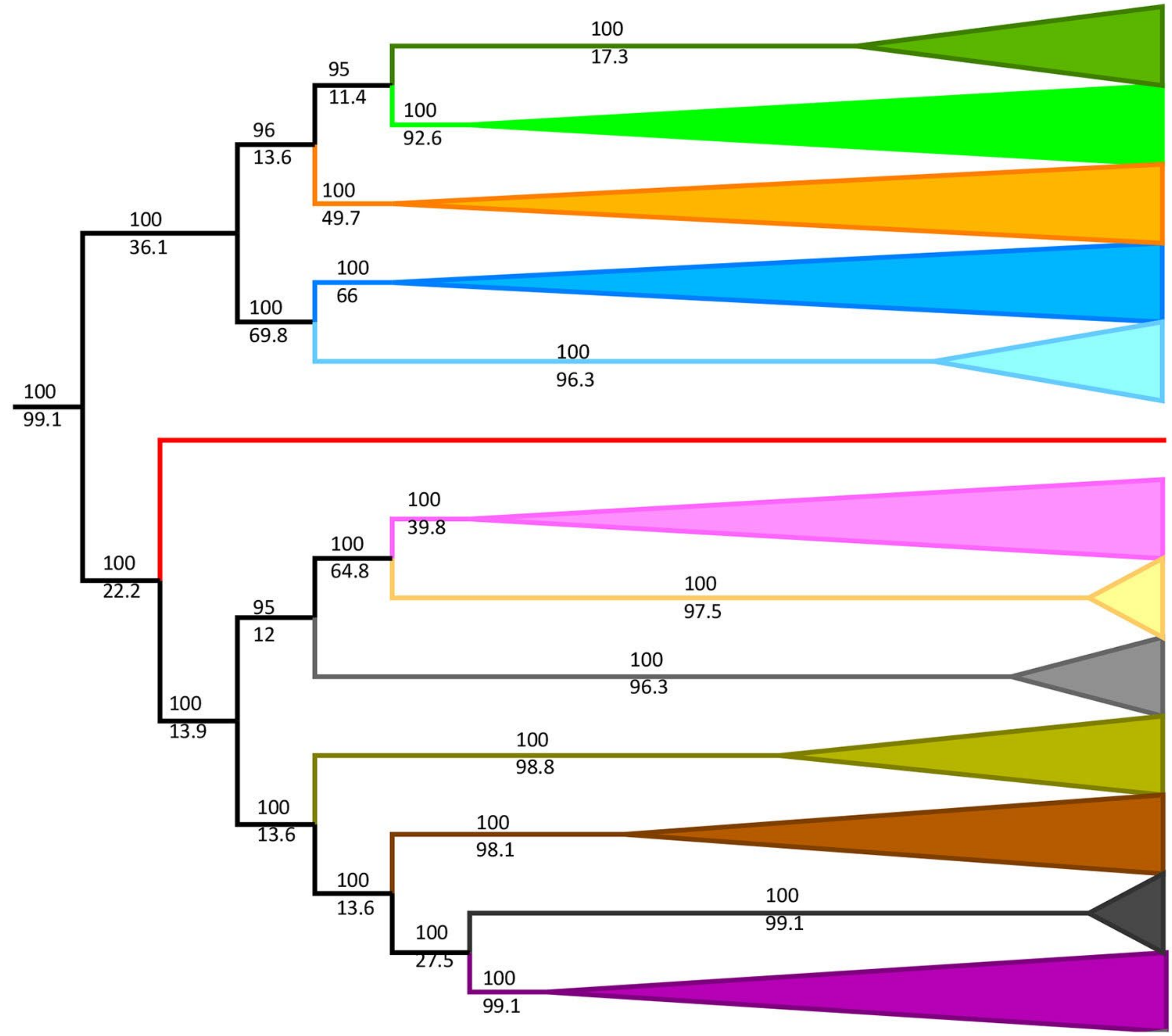

Appendix Figure 1. Branch support for each monophyletic group calculated with the 100kb genomic windows. Numbers on top of the branches are bootstrap support values; numbers under the branches are concordance factors. Lineages follow the same color scheme as Figures 1 and 2

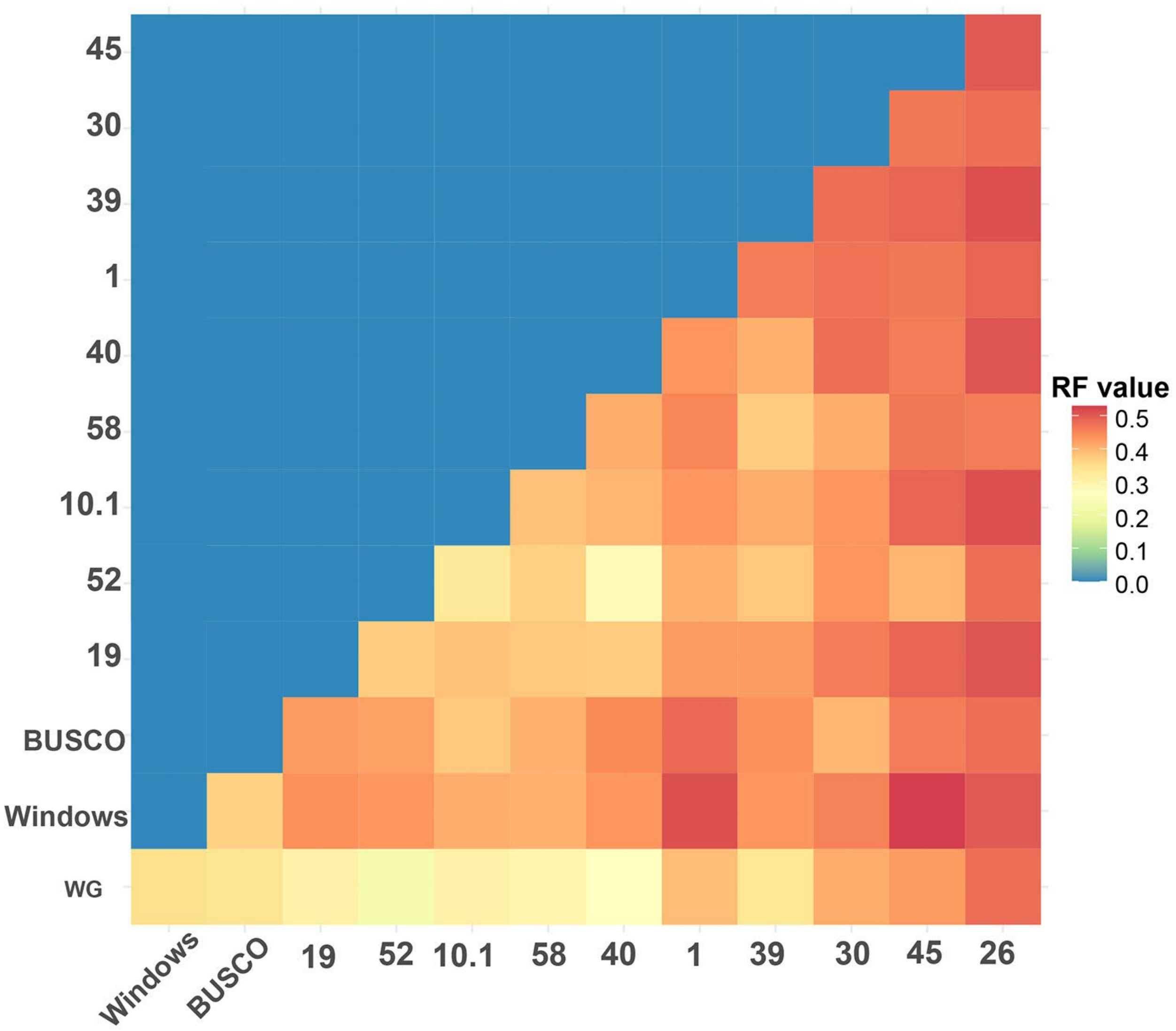

Appendix Figure 2. Robertson-Foulds (RF) distance between the topologies generated from different datasets, either each supercontig or the concatenated alignment.

References

1. Bolger AM, Lohse M, Usadel B. Trimmomatic: a flexible trimmer for Illumina sequence data. Bioinformatics. 2014;30:2114–20. PubMed https://doi.org/10.1093/bioinformatics/btu170

2. Li H, Durbin R. Fast and accurate short read alignment with Burrows-Wheeler transform. Bioinformatics. 2009;25:1754–60. PubMed https://doi.org/10.1093/bioinformatics/btp324

3. DePristo MA, Banks E, Poplin R, Garimella KV, Maguire JR, Hartl C, et al. A framework for variation discovery and genotyping using next-generation DNA sequencing data. Nat Genet. 2011;43:491–8. PubMed https://doi.org/10.1038/ng.806

4. McKenna A, Hanna M, Banks E, Sivachenko A, Cibulskis K, Kernytsky A, et al. The genome analysis toolkit: a mapreduce framework for analyzing next-generation DNA sequencing data. Genome Res. 2010;20:1297–303. PubMed https://doi.org/10.1101/gr.107524.110

5. Jofre GI, Singh A, Mavengere H, Sundar G, D'Agostino E, Chowdhary A, et al. An Indian lineage of *Histoplasma* with strong signatures of differentiation and selection. Fungal Genet Biol. 2022;158:103654. PubMed https://doi.org/10.1016/j.fgb.2021.103654

6. Almeida-Silva F, de Melo Teixeira M, Matute DR, de Faria Ferreira M, Barker BM, Almeida-Paes R, et al. Genomic diversity analysis reveals a strong population structure in *Histoplasma capsulatum* LAmA (*Histoplasma suramericanum*). J Fungi (Basel). 2021;7:865. PubMed https://doi.org/10.3390/jof7100865

7. Sepúlveda VE, Márquez R, Turissini DA, Goldman WE, Matute DR. Genome sequences reveal cryptic speciation in the human pathogen *Histoplasma capsulatum.* MBio. 2017;8:e01339–17. PubMed https://doi.org/10.1128/mBio.01339-17

8. Chowdhary A, Jofre GI, Singh A, Dagilis AJ, Sepúlveda VE, McClure AT, et al. Autochthonous *Blastomyces dermatitidis*, India. Emerg Infect Dis. 2024;30:2577–82. PubMed https://doi.org/10.3201/eid3012.240830

9. Muñoz JF, McEwen JG, Clay OK, Cuomo CA. Genome analysis reveals evolutionary mechanisms of adaptation in systemic dimorphic fungi. Sci Rep. 2018;8:4473. PubMed https://doi.org/10.1038/s41598-018-22816-6

10. Mavengere H, Mattox K, Teixeira MM, Sepúlveda VE, Gomez OM, Hernandez O, et al. *Paracoccidioides* genomes reflect high levels of species divergence and little interspecific gene flow. MBio. 2020;11:e01999–20. PubMed https://doi.org/10.1128/mBio.01999-20

11. Tenório BG, Kollath DR, Gade L, Litvintseva AP, Chiller T, Jenness JS, et al. Tracing histoplasmosis genomic epidemiology and species occurrence across the USA. Emerg Microbes Infect. 2024;13:2315960. PubMed https://doi.org/10.1080/22221751.2024.2315960
